# The impact of carbapenem shortage and stewardship countermeasures on antimicrobial practice at a tertiary care center

**DOI:** 10.1017/ash.2023.460

**Published:** 2023-10-19

**Authors:** Yuichi Kouyama, Yuki Uehara, Akane Takamatsu, Ryota Suzuki, Rie Nishida, Katsumasa Inuzuka, Eriko Muramatsu, Koji Ohyama, Yohei Doi, Shigeki Yamada, Hitoshi Honda

**Affiliations:** 1 Division of Antimicrobial Stewardship Program, Department of Quality and Safety in Healthcare, Fujita Health University School of Medicine, Toyoake, Aichi, Japan; 2 Department of Pharmacy, Fujita Health University Hospital, Toyoake, Aichi, Japan; 3 Department of Infectious Diseases, Fujita Health University School of Medicine, Toyoake, Aichi, Japan; 4 Graduate School of Public Health, St. Luke’s International University, Tokyo, Japan; 5 Division of Infection Control, Department of Quality and Safety in Healthcare, Fujita Health University School of Medicine, Toyoake, Aichi, Japan; 6 Division of Infectious Diseases, University of Pittsburgh School of Medicine, Pittsburgh, PA, USA

## Abstract

We evaluated the impact of carbapenem shortage on antimicrobial practice and patient outcome at a tertiary care center. During the shortage, hospital antimicrobial practice could be safely managed through additional antimicrobial stewardship measures including treatment guidance and mandatory preauthorization. Antimicrobial shortage may present a unique opportunity for promoting antimicrobial stewardship.

## Introduction

In recent years, shortages of essential medicines, including antimicrobial agents, have often caused challenges in clinical settings; therefore, the World Health Organization proposed the model list of essential medicines.^1^ While carbapenem antimicrobials are classified in the ‘Watch’ group in the list, they are frequently used for empiric therapy in nosocomial settings because of their broad-spectrum coverage, structural stability in the presence of most β-lactamases, and generally favorable safety profile.^2^ Because of their clinical importance, many antimicrobial stewardship programs (ASPs) have focused on judicious use of carbapenems to mitigate selective pressure by carbapenems that may induce resistance and ultimately compromise their clinical utility.^3–5^

In August 2022, a nationwide shortage of meropenem occurred in Japan because of contamination of the bulk powder of meropenem, which significantly limited the supply of all carbapenems.^6^ In response to the shortage, the study institution provided treatment guidance for presumed nosocomial infection and implemented mandatory preauthorization of all carbapenem antimicrobials (i.e., meropenem, imipenem–cilastatin, and doripenem). The present study aimed to assess the impact of the carbapenem shortage and subsequent ASP countermeasures on inpatient antimicrobial practice and in-hospital mortality.

## Methods

### Study setting

We conducted the observational study at Fujita Health University Hospital (FHUH), a 1376-bed tertiary care center in Japan. The antimicrobial stewardship team at FHUH consists of two infectious diseases (ID) physicians, two clinical pharmacists, two infection prevention nurses, and one clinical microbiology technologist. Prior to the carbapenem shortage, we performed postprescription review with feedback (PPRF) for patients receiving key antipseudomonal antimicrobials, including carbapenems, piperacillin–tazobactam, and fluoroquinolones for more than 14 d. Carbapenems on the formulary at FHUH included meropenem, imipenem–cilastatin, and doripenem but not ertapenem. Newer agents for multidrug-resistant gram-negative organisms (e.g., ceftazidime–avibactam, ceftolozane–tazobactam) were not formulary medications at the study institution.

### Antimicrobial stewardship countermeasures for the carbapenem shortage

On August 9, 2022, we received an advance notification of the meropenem shortage from the supplier. We provided treatment guidance for presumed nosocomial infections to prescribing physicians, endorsing prioritization of piperacillin–tazobactam and cefepime over meropenem (Supplementary Figure 1). On September 1, 2022, a mandatory preauthorization program was implemented for meropenem, which was expanded to include imipenem–cilastatin and doripenem on October 24, 2022. Under the program, use of carbapenems was allowed for patients with a history of infection by extended-spectrum or AmpC-β-lactamase-producing Enterobacterales or those who were clinically failing treatment with cefepime or piperacillin–tazobactam. In the absence of either of the above conditions, consultation with an ID physician was mandated before a carbapenem could be ordered.

**Figure 1.**
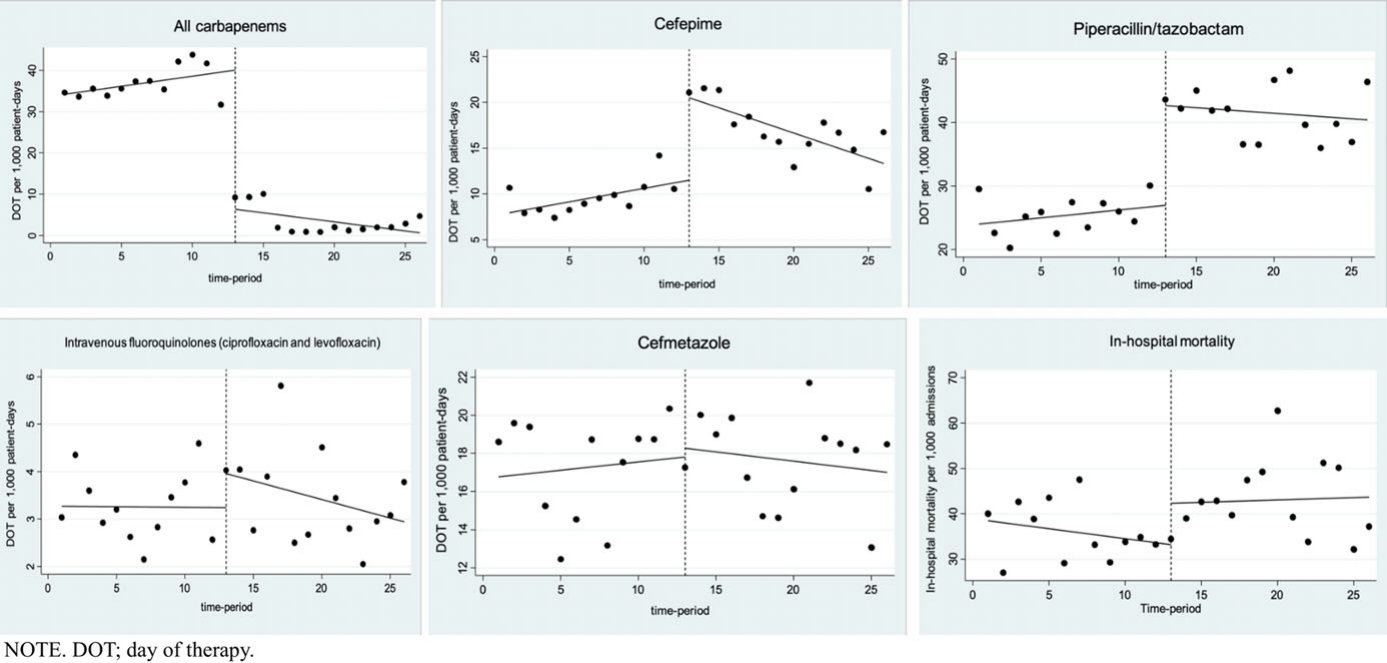
Antimicrobial use and in-hospital mortality before and during the carbapenem shortage.

### Data collection and statistical analysis

Use of all intravenous antimicrobials including carbapenem, expressed as days of therapy per 1,000 patient-days, and inpatient mortality rate, was collected fortnightly from March 2022 through February 2023. In the present study, we assessed changes in the trend on the use of antipseudomonal agents (i.e., carbapenems, cefepime, piperacillin–tazobactam, fluoroquinolones) and cefmetazole (which was frequently used for treating infections due to extended-spectrum β-lactamase-producing organisms).^7^ As a patient-related outcome, the in-hospital mortality rate was tracked as the number of deaths per 1,000 new admissions every 2 weeks. We used the segmented regression in interrupted time-series analysis (ITSA) to assess the differences in antimicrobial use and inpatient mortality rate between the preshortage period (from March to August 2022) and the shortage period (from September to March 2023). The study was approved by the institutional review committee at FHUH.

## Results

Figure 1 shows the use of each broad-spectrum antimicrobial agent before and after the carbapenem shortage as determined by ITSA. Overall carbapenem (meropenem, imipenem–cilastatin, and doripenem) use significantly decreased at an intercept (−33.67 DOT per 1,000 patient-days [PD], *P* < 0.001), whereas the change in the slope was not statistically significant (−0.43 DOT per 1,000 PD, *P* = 0.12). In response to the treatment guidance and carbapenem preauthorization, the use of alternative antipseudomonal agents, including piperacillin–tazobactam and cefepime, substantially increased at the intercept level (Table 1). Changes in the use of other alternative antimicrobial agents at the intercept and trend levels are shown in Table 1 and Figure 1. Regarding changes in in-hospital mortality rate, a higher in-hospital mortality rate at the intercept level was observed (+9.09 per 1,000 admissions; *P* = 0.04), whereas that in trend level was steady during the carbapenem shortage period (+0.10 per 1,000 admissions; *P* = 0.85). The acceptance rate of PPRF increased after the carbapenem shortage occurred. Details of PPRF are shown in Supplementary Table 1. The mean number of ID consultations during the study period was 48 (range: 36–67), shown in Supplementary Table 2.

**Table 1.** Changes in antimicrobial use and in-hospital mortality rate after the carbapenem shortage and implementation of antimicrobial stewardship countermeasures

Outcomes	Changes at an intercept (95% CI)	*P* value	Changes in a trend (95% CI)	*P* value
**Antimicrobial use (DOT per 1,000 patient-days)**				
Carbapenem	−33.67 (−40.90, −26.44)	<0.001	−0.43 (−0.97, +0.10)	0.11
Piperacillin/tazobactam	+15.69 (+13.29, +18.08)	<0.001	−0.17 (−0.46, +0.11)	0.23
Cefepime	+9.01 (+7.03, +11.00)	<0.001	−0.55 (−0.78, −0.32)	<0.001
Intravenous fluoroquinolones	+0.71 (−0.25, +1.68)	0.14	−0.08 (−0.13, −0.02)	0.009
Cefmetazole	+0.46 (−2.78, +3.69)	0.77	−0.10 (−0.29, +0.09)	0.29
**Patient outcome**				
In-hospital mortality rate (number of deaths per 1,000 admissions)	+9.09 (+0.49, +17.69)	0.04	+0.10 (−1.03, +1.23)	0.85

Note. CI, confidence interval; DOT, day of therapy.

## Discussion

The significant shortage of carbapenems owing to manufacturing issues did not substantially impact overall broad-spectrum antimicrobial use at the study institution. Moreover, the carbapenem shortage provided an unexpected opportunity to rapidly trigger a behavioral change in prescription away from carbapenems toward alternative broad-spectrum agents.

As shown in Figure 1, carbapenem use significantly decreased at the intercept level after the carbapenem shortage occurred and remained consistently low under the shortage and mandatory preauthorization. Use of the substitute agents increased substantially as a squeezing balloon effect at the intercept level, but their use gradually decreased over time as the carbapenem shortage continued. This favorable change during the carbapenem shortage was likely multifactorial. The empiric antimicrobial use guidance may have helped physicians choose an appropriate empiric therapy on their own and more comfortably. Since the mandatory carbapenem preauthorization by the ASP was started immediately after the carbapenem shortage occurred, this may have further promoted appropriate antimicrobial use of not only carbapenems but also piperacillin–tazobactam, cefepime, and fluoroquinolones. As seen in Supplementary Table 2, the monthly numbers of formal ID consultation were stable overtime. Providers also made inquiries in the form of curbside ID and AST consultation especially regarding empiric antimicrobial choice, but these numbers were not tracked.

The preexisting PPRF for prolonged use of carbapenems and piperacillin–tazobactam at the study institution may have also synergistically optimized piperacillin–tazobactam and cefepime use. We hypothesize that curbside ID and AST consultation in conjunction with preexisting PPRF were leveraged to ensure antimicrobial management, advise treating physicians of optimal duration of therapy, and promote early de-escalation. PPRF is also an essential strategy to mitigate the detrimental effects of the broad-spectrum antimicrobial shortage.^8^

Association between in-hospital mortality and carbapenem shortage at the intercept level by ITSA was observed, which is a potential concern as the finding may suggest that the lack of access to the broadest-spectrum antimicrobials resulted in a temporary increase in mortality at the study institution. However, a review of medical records revealed that the proportion of in-hospital deaths due to infections only susceptible to carbapenem antimicrobials and for which carbapenems would conventionally be considered to be first-line agents was stable before and after the carbapenem shortage occurred (Supplementary Table 3). Additionally, an intermittent increase in in-hospital mortality was also observed from early November to late December in 2022; the cause of death in these patients included end-stage illnesses due to malignancies, COVID-19, and cerebrovascular accidents but not due to healthcare-associated infection. Whereas the findings in the present study suggest that patient safety may not be compromised by limited availability of carbapenems, ASP countermeasures for antimicrobial shortage should still be implemented to mitigate an impact of the antimicrobial shortage on patient outcome since drug shortage may cause a substantial increase in the use of alternative antimicrobials and the incidence of *Clostridioides difficile* infection in previous reports.^9,10^

The present study has some limitations. Because this was a single-center study, the described approach may not be applicable to other institutions. Since the carbapenem shortage only lasted 7 months, changes in antimicrobial susceptibilities or the incidence of MDRO infections, especially those due to gram-negative bacteria and *C. difficile*, could not be ascertained. Lastly, the upsurge of COVID-19, which occurred from December 2022 to January 2023, may have impacted antimicrobial consumption.

In conclusion, promotion of non-carbapenem agents as empiric antimicrobial therapy for presumed nosocomial infection and mandatory preauthorization for carbapenem use, implemented in response to a significant carbapenem shortage, contributed to improved overall antimicrobial practice. Drug shortage may thus provide a unique opportunity to advance antimicrobial stewardship objectives.

## Supporting information

Kouyama et al. supplementary material Kouyama et al. supplementary material

## Supplementary material

For supplementary material accompanying this paper visit http://doi.org/10.1017/ash.2023.460. click here to view supplementary material
