# Supplementary material for: The impact of carbapenem shortage and stewardship countermeasures on antimicrobial practice at a tertiary care center
Source: Antimicrob Steward Healthc Epidemiol. 2023 Oct 19;3(1):e173. doi: 10.1017/ash.2023.460 (PMC10644165; doi:10.1017/ash.2023.460)
Supplement: Kouyama et al. supplementary material [file S2732494X23004606sup001.docx]

**Supplementary file**

**Supplementary Table 1.** Details of post-prescription review and feedback during the study period.

|  | Mar 2022 | April 2022 | May 2022 | June 2022 | July 2022 | Aug 2022 | Sep 2022 | Oct  2022 | Nov 2022 | Dec 2022 | Jan  2023 | Feb 2023 | Mar 2023 | Apr 2023 |
| --- | --- | --- | --- | --- | --- | --- | --- | --- | --- | --- | --- | --- | --- | --- |
| Number of PPRF | 7 | 18 | 16 | 32 | 24 | 20 | 22 | 27 | 12 | 23 | 22 | 12 | 30 | 23 |
| Overall acceptance rate  (%) | 2/7  (29%) | 2/18  (11%) | 5/16  (31%) | 7/32  (22%) | 10/24  (42%) | 14/20  (70%) | 14/22  (64%) | 18/27  (67%) | 8/12  (67%) | 13/23  (57%) | 15/22  (68%) | 8/12  (67%) | 22/30  (73%) | 13/23  (57%) |
| Recommendation from antimicrobial stewardship program | | | | | | | | | | | | | | |
| De-escalation | 2 | 2 | 1 | 6 | 2 | 6 | 4 | 12 | 4 | 8 | 5 | 3 | 10 | 2 |
| Escalation | 2 | 1 | 0 | 7 | 1 | 3 | 2 | 3 | 3 | 2 | 3 | 1 | 3 | 4 |
| Obtain repeat culture | 1 | 7 | 3 | 6 | 6 | 4 | 5 | 2 | 0 | 2 | 2 | 0 | 7 | 5 |
| Dose adjustment | 1 | 2 | 5 | 1 | 1 | 1 | 3 | 1 | 1 | 4 | 2 | 0 | 1 | 2 |
| Stop antibiotics | 1 | 6 | 7 | 12 | 14 | 6 | 8 | 9 | 4 | 7 | 1 | 8 | 9 | 1 |

NOTE. PPRF; post-prescription review and feedback

**Supplementary table 2.** Number of infectious diseases consultation during the study period

|  | Mar 2022 | April 2022 | May 2022 | June 2022 | July 2022 | Aug 2022 | Sep 2022 | Oct 2022 | Nov 2022 | Dec 2022 | Jan 2023 | Feb 2023 | Mar 2023 | Apr 2023 |
| --- | --- | --- | --- | --- | --- | --- | --- | --- | --- | --- | --- | --- | --- | --- |
| Number of ID consultation | 59 | 36 | 43 | 43 | 50 | 50 | 67 | 37 | 41 | 52 | 57 | 40 | 44 | 54 |

NOTE. ID; infectious diseases

**Supplementary Table 3.** Number of in-hospital death at the time of carbapenem shortage.

|  | 06/2022 | 07/2022 | 08/2022 | 09/2022 | 10/2022 | 11/2022 | 12/2022 |
| --- | --- | --- | --- | --- | --- | --- | --- |
|  | Pre-carbapenem shortage period | | | Beginning of carbapenem shortage | Carbapenem shortage period | | |
| Total number of in-hospital death | 112 | 86 | 92 | 92 | 112 | 116 | 135 |
| In-hospital death due to infections only susceptible to carbapenem antimicrobials, and infections for which carbapenems were conventionally considered  to be first-line agents. | 4 (3.6%) | 1 (1.2%) | 1(1.1%) | 0 (2.2%) | 2 (1.8%) | 2 (1.7%) | 2 (1.5%) |

**Supplementary Figure 1.**

Alternative empiric antimicrobial therapy recommendation for carbapenems at Fujita Health University Hospital


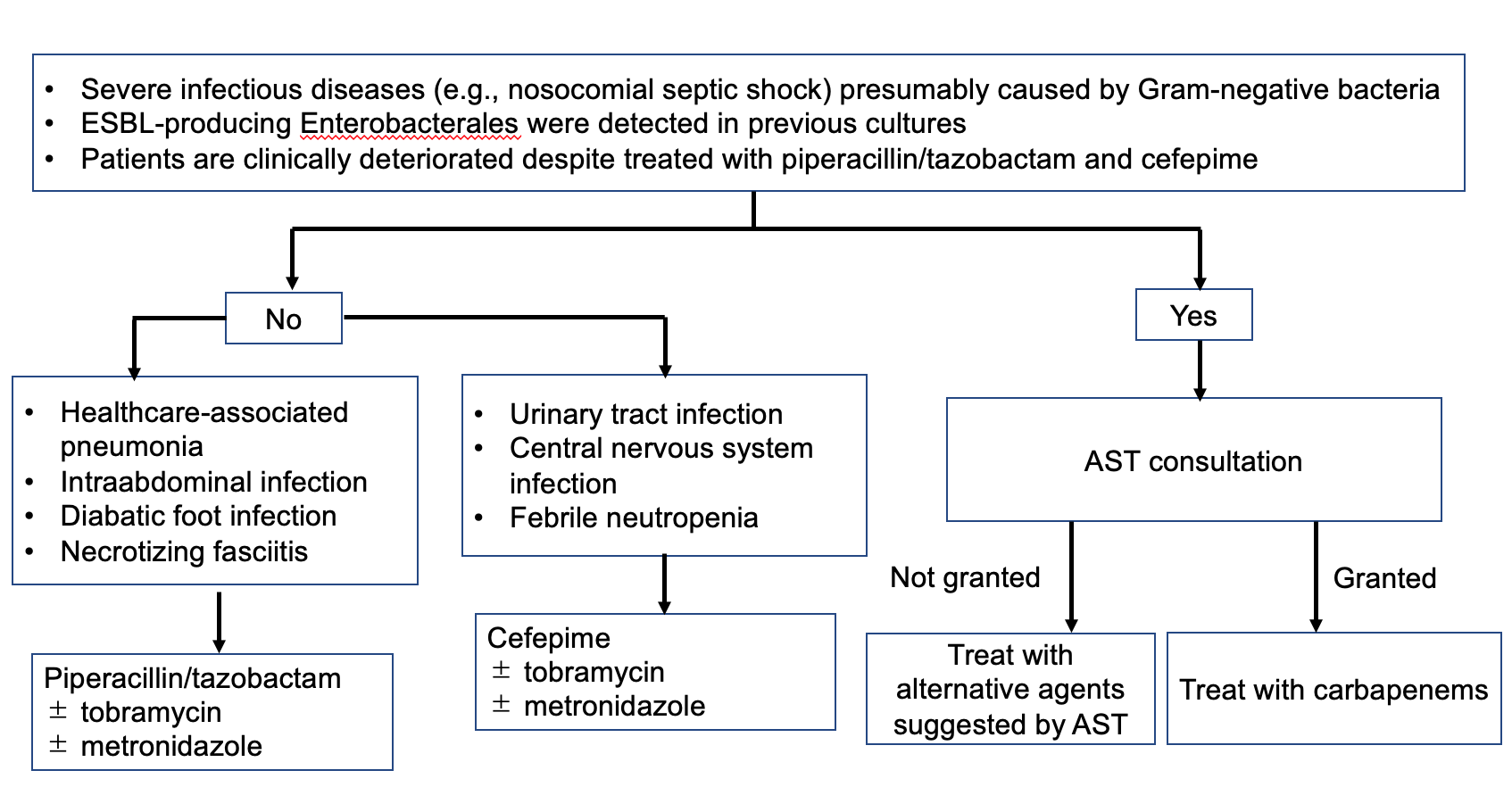


NOTE. ESBL; extended-spectrum β-lactamase, AST; antimicrobial stewardship team
